# Fine mapping of new glaucoma locus *GLC1M* and exclusion of *neuregulin 2* as the causative gene

**Published:** 2007-05-23

**Authors:** Bao Jian Fan, Wendy Charles Ko, Dan Yi Wang, Oscar Canlas, Robert Ritch, Dennis S. C. Lam, Chi Pui Pang

**Affiliations:** 1 Department of Ophthalmology and Visual Sciences, The Chinese University of Hong Kong, Hong Kong, China; 2 Jose B. Lingad Memorial Regional Hospital, City of San Fernando, Philippines; 3 Departments of Ophthalmology, the New York Eye and Ear Infirmary, New York, and the New York Medical College, Valhalla, NY

## Abstract

**Purpose:**

We recently identified a novel glaucoma locus on 5q22.1-q32, designated as *GLC1M*, in a family from the Philippines with autosomal dominant juvenile-onset primary open angle glaucoma (JOAG). No mutations in *myocilin* (*MYOC*), *optineurin* (*OPTN*), and *WD-repeat protein 36* (*WDR36*) were found. *Neuregulin 2* (*NRG2*) is an excellent potential functional as well as positional candidate at *GLC1M*. The goal of the present study was to evaluate the role of the *NRG2* gene in this JOAG family and unrelated JOAG patients and to refine the critical interval for *GLC1M*.

**Methods:**

Genomic DNA was obtained from 27 family members. All coding exons and splicing sites of *NRG2* were screened for sequence alterations by polymerase chain reaction (PCR) and DNA sequencing. A cohort of 92 unrelated JOAG patients and 92 control subjects were genotyped for the three single nucleotide polymorphisms (SNPs) of *NRG2* by PCR and DNA sequencing. Haplotype and segregation analyses were performed in the family. Fisher's exact test was used to compare the frequencies of the *NRG2* polymorphisms between affected and unaffected subjects in the family and between unrelated JOAG patients and control subjects.

**Results:**

Three SNPs were identified: c.98G>A (S33N), IVS3+13A>G (rs889022), and c.1976A>G (G659G). None of them segregated with the JOAG phenotype in this family. No association was found between *NRG2* and JOAG in the case-control study (p>0.12). However, further inspection of the haplotypes in the family localized the *NRG2* gene telomeric to the disease locus. The critical interval of *GLC1M* was therefore refined to a region of 28 Mb between D5S2051 and *NRG2*.

**Conclusions:**

The linkage interval for *GLC1M* was refined to a smaller region. The *NRG2* gene was excluded as the causative gene for JOAG.

## Introduction

Primary open angle glaucoma (POAG; OMIM 137760) is the most common form of glaucoma, affecting over 33 million people worldwide. It is a progressive optic neuropathy characterized by a specific pattern of cupping of the optic disc with correspondent visual field loss and is potentially blinding [1]. There are two types of POAG: juvenile-onset POAG (JOAG) and adult-onset POAG. By definition, JOAG develops before 35 years of age [2,3] and is typically inherited as an autosomal-dominant trait, whereas adult-onset POAG is inherited as a complex trait [4]. Elevated intraocular pressure (IOP; greater than or equal to 22 mmHg) is the most common known risk factor for POAG [5]. However, approximately 25% of patients have an IOP level lower than this reference level and are considered to have normal-tension glaucoma (NTG) [6] or low-tension glaucoma [7].

POAG is genetically heterogeneous, with links to at least 22 genetic loci [8,9]. Among them, 14 loci designated *GLC1A* to *GLC1N* have been defined for POAG using family-based linkage studies. So far, three genes have been identified for POAG from the reported genetic loci: *myocilin* (*MYOC*, OMIM 601652) [10,11], *optineurin* (*OPTN*, OMIM 602432) [12], and *WD repeat-domain 36* (*WDR36*, OMIM 609669) [13]. Only *MYOC* has been established as directly causative of glaucoma [14-19], while the role of *OPTN* is still unclear due to conflicting evidence [19-23] and *WDR36* is considered to be a modifier gene for glaucoma [24-27]. Mutations in these three genes account for no more than 10% of POAG cases [8]. Moreover, at least 16 POAG-associated genes have been reported from association studies [8]. There is discrepancy in the reported roles of these genes in the etiology of POAG. It is therefore evident that additional loci or genes are involved in the development of POAG.

Recently, we mapped a novel JOAG locus to 5q22.1-q32 in a large autosomal-dominant JOAG family from the Philippines [24]. This five-generation family had a total of 95 members, 22 of whom were affected with JOAG. Complete ophthalmic examination was given to 27 family members, in which nine were confirmed JOAG patients [28]. After exclusion of *MYOC* and *OPTN* as disease-causing genes in this family, a genome-wide search was carried out using 382 microsatellite markers with average spacing of 10 cM. Fine mapping and haplotype analysis identified a new JOAG locus at 5q22.1-q32 within a region of 36 Mb flanked by D5S2051 and D5S2090, designated as *GLC1M* (OMIM 610535) by the HUGO gene nomenclature committee. This JOAG locus did not overlap with the *GLC1G* minimal interval between D5S1466 and D5S2051 [13]. However, discrepancy between the genetic and physical maps may still position the *WDR36* gene, located at *GLC1G*, within our disease interval. We therefore screened *WDR36* for mutations in affected family members. No sequence variations in the coding exons or splicing junctions of *WDR36* were found to be associated with glaucoma. Although we could not rule out possible variations within the introns or the promoter of *WDR36*, our data strongly suggested the presence of an independent JOAG gene on 5q. Further search for the causative gene at *GLC1M* is warranted.

A candidate gene, *neuregulin 2* (*NRG2*, OMIM 603818), located at 5q23-q33, was within the critical region of *GLC1M* [29]. NRG2 is a member of neuregulins that are a family of growth and differentiation factors related to epidermal growth factor. Through interaction with the ErbB receptors, neuregulins induce the growth and differentiation of epithelial, neuronal, glial, and other types of cells [30]. In particular, it has been demonstrated that neuregulin-ErbB signaling pathways play crucial roles in regulating the proliferation and differentiation of Schwann cells, which are the myelin-forming cells in the peripheral nervous system. NRG2 has been identified as a factor capable of promoting the subventricular zone proliferation, leading to the formation of new neurons [31]. NRG2 promotes GFAP^+^ cell proliferation and polysialylated neural cell adhesion molecule (PSA-NCAM^+^) neuroblast generation [31]. Although the fundamental pathophysiology of glaucoma is largely unknown, it is believed that retinal ganglion cell death is the ultimate pathway. Glaucoma is thus considered a disorder of optic nerve degeneration. *NRG2* is therefore an excellent potential functional as well as positional candidate gene for glaucoma at *GLC1M*. In the present study, we evaluated the role of the *NRG2* gene in the JOAG family from whom the *GLC1M* locus was originally identified and unrelated JOAG patients, and further refined the linkage interval for *GLC1M*.

## Methods

### Description of family with JOAG

As previously reported, a large family was recruited from the Ibanez region of the Philippines [28]. Two ophthalmologists (Drs. Canlas O. and Ritch R.) examined the family members and evaluated the whole family. The study protocol was approved by the Ethics Committee for Human Research, the Chinese University of Hong Kong. In accordance with the tenets of the Declaration of Helsinki, informed consent was obtained from all participants after explanation of the nature and possible consequences of the study. This five-generation family had a total of 95 members, in which 22 were affected with JOAG. Complete ophthalmic examination was given to 27 family members, nine of whom were confirmed JOAG patients. Peripheral venous whole blood from these subjects was collected for genomic DNA extraction. The other family members did not agree to participate in this study. Their clinical information was obtained through previous medical records. A definition of JOAG was based on the following criteria: exclusion of secondary causes (e.g., trauma, uveitis, or steroid-induced glaucoma), Shaffer grade III or IV open iridocorneal angle on gonioscopy, IOP greater than or equal to 22 mmHg in both eyes by applanation tonometry, characteristic optic disc damage or typical visual field loss by Humphrey automated perimeter with the Glaucoma Hemifield test, and diagnosis before age 35. For the affected subjects, age at diagnosis ranged from 12 to 33 years (mean±SD: 19±4.2 years), the highest IOP from 24 to 44 mmHg (mean±SD: 32±6.3 mm Hg), vertical cup-disc ratio from 0.7 to 0.9 (mean±SD: 0.8±0.04), and visual field loss was compatible with glaucoma in two consecutive Humphrey testing. For the unaffected subjects, age at inclusion ranged from 3 to 73 years (mean±SD: 25±19.9 years), IOP<22 mmHg, vertical cup-disc ratio from 0.2 to 0.5 (mean±SD: 0.3±0.06), and visual field was in normal range.

### Unrelated juvenile open angle glaucoma patients and controls

A cohort of 92 unrelated patients with JOAG and 92 unrelated control subjects without glaucoma were genotyped for the 3 *NRG2* polymorphisms identified from the JOAG family. The unrelated JOAG group was comprised of 54 males and 40 females. Their age at diagnosis ranged 8-34 years (mean±SD: 25±5.4 years), the highest IOP was 23-50 mmHg (mean±SD: 29±5.9 mm Hg), vertical cup-disc ratio was 0.7-0.9 (mean±SD: 0.8±0.05), and their visual field loss was compatible with glaucoma in two consecutive Humphrey testing. The control group had 51 males and 43 females, whose age at inclusion ranged 60-83 years (mean±SD: 73±3.8 years), IOP<22 mmHg, and whose vertical cup-disc ratio was 0.1-0.5 (mean±SD: 0.3±0.07), visual fields within normal range, and had no family history of glaucoma.

### Mutation screening

Genomic DNA was extracted from 200 μl of blood using a commercial kit (Qiamp Blood Kit; Qiagen, Hilden, Germany). Quantification of extracted DNA was performed using NanoDrop ND-1000 spectrophotometer (NanoDrop Technologies, Wilmington, DE). The coding exons and splicing sites of *NRG2* were amplified by polymerase chain reaction (PCR), followed by DNA sequencing. Primers used to obtain the initial amplicons are given in Table 1. Initial PCRs were performed on a thermal cycler (model 9700; Applied Biosystems [ABI], Foster City, CA) in a total volume of 25 μl containing 200 ng of genomic DNA, 0.4 μM of each primer, 200 μM dNTPs, 20 mM Tris-HCl (pH 8.0), 50 mM KCl, 1.5 to 3.0 mM MgCl_2_, and 1 U of *Taq* DNA polymerase (Ampli*Taq* Gold; ABI). Cycling conditions were as follows: first denaturation step of 12 min at 94 °C, 35 cycles of denaturation (94 °C for 40 s), annealing (primer-specific annealing temperature for 60 s), elongation (72 °C for 40 s), and a final single elongation step of 7 min. The PCR products were electrophoresed on 2% agarose gel and visualized using a video gel documentation system (Gel-Doc 2000; BioRad Laboratories, Hercules, CA) to check for the quality. The PCR products were then purified with ExoI-SAP kit (USB Corp., Cleveland, OH) to remove unconsumed dNTPs and primers. A second PCR was performed using the sequencing primers as described in Table 1 on a thermal cycler (model 9700; ABI) to incorporate the sequencing dyes (BigDye® Terminator v3.1 cycle sequencing kit; ABI) using a protocol of 25 cycles of denaturation (96 °C for 10 s), annealing (50 °C for 5 s), and elongation (60 °C for 4 min). Sequence data were then aligned using Sequence Navigator analysis software (version 1.0.1; ABI) and compared with the published *NRG2* gene sequence (GenBank AH009107).

**Table 1:** Polymerase chain reaction primers and conditions for *NRG2* mutation screening.

**Primer**	**Primer sequence**	**Amplicon size (bp)**	**Mg^2+^ concentration (mM)**	**Annealing temperature (°C)**
1AF	TTTCCGGTTTTCCAGCGGG	408	3.0	60
1AR	CTGTGTGGCTTCTCGTCGTACC			
1AF	TTTCCGGTTTTCCAGCGGG	783	1.5	64
1BR	GGCGTCAGTCACGTGTCCTAG			
1BF	TTCGCGAGCCGCAGCC	450	3.0	62
1BR	GGCGTCAGTCACGTGTCCTAG			
2F	CCTTACTCTCCACTACTCATGCTTGGC	284	1.5	60
2R	TCGACGAACCTACCTCCTGTCCG			
3F	TGGAGAGAGGCAACCGCTGG	188	1.5	60
3R	GTTTGGGGAGATCCTGGGAAGGG			
4F	GCATGAAGGAGATGATTCCTGGG	231	1.5	60
4R	AAGAACGAGGGTACGGGTGG			
5F	TCAGCTACAAGTATGACCCCAAGTGC	128	1.5	60
5R	GGGTCTTCGAAAGATTCCTCGTCC			
6F	ATGGTAACGGTGGCAAGGAACC	310	1.5	60
6R	CCACAAAGGCAGAGGAGATTCCTCG			
7F	TGTCTGAGGAGTCCTGACCAACG	186	2.0	58
7R	CTCCCCGGTGCGTCTACC			
8F	GGTCTCTGCACCACTATCCCTATGG	233	1.5	58
8R	GTGTAAGAACCTCCGGGTAGG			
9F	AGATAGCTAGGGAAGTTCATCGTTGG	251	1.5	58
9R	GGACAGCCAGCCTTCTCATGC			
10F	AACAAGAAAGAGTTCATTTGGGCCC	222	1.5	58
10R	GATGTTCAAAGGTACCCGGAACC			
11F	CATTGAGCTAAGGGAGCTCGAGG	393	3.0	58
11R	ACGGTCGGGAGACCGATTCG			
12AF	TGGCCCATGCCTCTGCC	1107	2.0	60
12BF	CCGAGGACGACGAGTACGAGA			
12AR	GAGACCAGAGGAATTTCTATCACCCCG			

Primers used to obtain the initial PCR amplicons and for subsequent sequencing of the *NRG2* gene are listed in this table. Exon 1 was split into three amplicons for PCR and sequencing. Exon 12 was initially amplified by PCR using primers 12AF and 12AR and subsequently sequenced using primers 12AF and 12BF.

Statistical analyses were performed using SAS statistical software (version 9.1.3; SAS Institute, Cary, NC). Fisher's exact test was used to compare the frequencies of the *NRG2* polymorphisms between affected and unaffected subjects in the family and between unrelated JOAG patients and controls.

## Results

### Evaluation of *NRG2* as a candidate gene in *GLC1M* for juvenile open angle glaucoma

**Figure 1:**
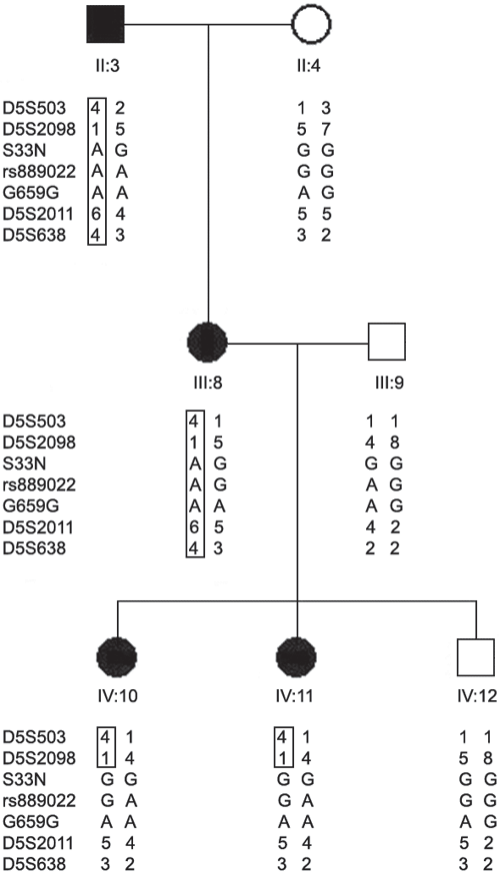
Haplotypes of markers flanking *GLC1M*. Based on the published pedigree structure [24], only seven family members who are informative for refinement of the critical region of *GLC1M* were included in this figure. Squares denote male family members while circles indicate females. Shaded shapes are family members with juvenile open angle glaucoma. Markers S33N, rs889022, and G659G are single nucleotide polymorphisms of *NRG2*.A rectangle encases segregating haplotypes. The haplotype for subject III:9 was inferred by using known genotypes from her offspring and husband.

As previously reported [28], the five generations of vertical inheritance of the JOAG phenotype displayed a direct male-to-male transmission with similar numbers of affected males and females. It was consistent with an autosomal-dominant pattern of inheritance. We screened a total of 27 subjects (nine with JOAG) for sequence alterations in the coding regions and splicing sites of *NRG2*. No disease-causing mutation was identified in *NRG2* in the JOAG family. Instead, three single nucleotide polymorphisms (SNPs) were found: one nonsynonymous SNP c.98G>A (S33N), one noncoding SNP IVS3+13A>G (rs889022), and one synonymous SNP c.1976A>G (G659G). S33N was found in 25.9% (7/27) of the subjects in the family, rs889022 in 40.7% (11/27) of the subjects, and G659G in 11.1% (3/27) of the subjects (Table 2). However, none of these SNPs segregated with the JOAG phenotype in this family (Figure 1).

**Table 2:** *NRG2* polymorphisms identified in a family with juvenile open angle glaucoma.

**Sequence change**	**Codon change**	**Location**	**Minor allele frequency**		**Genotype frequency**	
			**Affected (n = 18)**	**Unaffected (n = 36)**	**Affected (n = 9)**	**Unaffected (n = 18)**
c.98G>A	S33N	Exon 1	5 (0.28)*	2 (0.06)	0/5/4*	0/2/16
IVS3+13A>G (rs889022)	-	Intron 3	6 (0.33)	7 (0.19)	0/6/3*	2/3/13
C.1976A>G	G659G	Exon 10	0(0)	3 (0.08)	0/0/9	0/3/15

Fisher's exact test was used to compare the frequencies of the *NRG2* polymorphisms between affected and unaffected subjects. The asterisk indicates a p<0.05. However, no polymorphism was segregated with the juvenile open angle glaucoma phenotype in the family.

To evaluate the role of *NRG2* on unrelated patients with JOAG, we genotyped the three SNPs (S33N, rs889022, and G659G) in a cohort of 92 unrelated patients with JOAG and 92 unrelated control subjects without glaucoma. S33N and G659G were found to be wild-type in all subjects. rs889022 was found in 13.0% (12/92) of the JOAG patients and in 22.8% (21/92) of the control subjects (p=0.12). No association was found between *NRG2* and JOAG (p>0.12; Table 3).

**Table 3:** *NRG2* polymorphisms in unrelated patients with juvenile open angle glaucoma and controls.

**Sequence change**	**Codon change**	**Location**	**Minor allele frequency**		**Genotype frequency**	
			**JOAG** **(n = 184)**	**Control** **(n = 184)**	**JOAG** **(n = 92)**	**Control (n = 92)**
c.98G>A	S33N	Exon 1	0(0)	0(0)	0/0/92	0/0/92
IVS3+13A>G (rs889022)	-	Intron 3	14 (0.08)	23 (0.13)	2/10/80	2/19/71
C.1976A>G	G659G	Exon 10	0(0)	0(0)	0/0/92	0/0/92

Fisher's exact test was used to compare the frequencies of the *NRG2* polymorphisms between patients with juvenile open angle glaucoma (JOAG) and controls. No association was found between *NRG2* and JOAG. A p>0.12 was obtained for all three polymorphisms.

### Refinement of the *GLC1M* locus using intronic polymorphisms of *NRG2*

**Figure 2:**
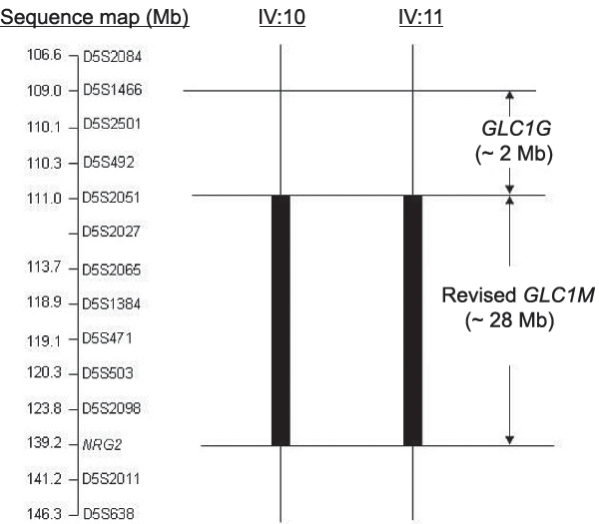
Recombination mapping of *GLC1M*. Solid rectangles indicate the nonrecombinant region for each individual. Horizontal lines mark the critical recombination event. The juvenile open angle glaucoma locus at *GLC1M* was revised centromerically at D5S2051 and telomerically at *NRG2*, within a region of 28 Mb. The revised *GLC1M* locus, while close to the *GLC1G* minimal interval, does not overlap it.

Haplotype analysis of the three SNPs of *NRG2* in the family with JOAG confirmed the recombination event in two affected individuals (IV:10 and IV:11, Figure 1). The *NRG2* gene was therefore placed telomeric to the disease locus. The critical interval of *GLC1M* was further refined to a region of 28 Mb between D5S2051 and *NRG2* (Figure 1 and Figure 2).

## Discussion

In the present study, we identified three SNPs in *NRG2*. The SNP rs889022 has been previously reported as a common polymorphism [29], while S33N and G659G are novel. None of these SNPs segregated with the JOAG phenotype in the family. No association was found between *NRG2* and JOAG in the case-control association study involving unrelated JOAG patients and controls. The *NRG2* gene was therefore excluded as the causative gene for JOAG. It indicates that an unidentified gene is associated with glaucoma in this family. Further inspection of the haplotypes of these SNPs in the family localized the *NRG2* gene telomeric to the disease locus. When we reanalyzed the original genotype data, we clarified the genotypes with respect to two markers, D5S2011 and D5S638, for individuals IV:10 and IV:11 (Figure 1). With the correct haplotypes of these two markers together with the haplotypes of 3 SNPs in *NRG2*, we redefined the critical region of *GLC1M* between D5S2051 and *NRG2* (Figure 2). The linkage interval of *GLC1M* was therefore refined to a smaller region of 28 Mb compared to the originally reported interval of 36 Mb [24].

As the revised candidate interval of *GLC1M* still covers a large distance of 28 Mb, the region can be further refined by recruiting more family members and genotyping more genetic markers. This, in turn, will be helpful in discovering the disease-causative gene. Besides *NRG2*, we also screened the *secreted protein acidic and rich in cysteine* gene (*SPARC*, OMIM 182120) but found no mutations in this JOAG family (data not shown). Although both genes are considered excellent potential functional as well as positional candidates at *GLC1M*, our work demonstrated that the candidate gene screening process is inefficient and is limited in its ability to identify disease-causative genes. We therefore attempted an alternative approach to better identify the disease-causative genes in linkage loci. We learned the principle from genome-wide association study [32], although our intention was not to investigate the whole genome, but instead a limited region of the genome, e.g., 15q22-q24 (*GLC1N*), where another JOAG locus was recently mapped within a genetic distance of 16.6 Mb [9]. To do that, we selected more than 100 gene-based SNPs within *GLC1N*, roughly one SNP for one gene. We genotyped these SNPs in a cohort of 100 unrelated JOAG patients and 100 control subjects. Several genes demonstrated significant association with JOAG in this case-control regional association study. These genes will be a priority in the search for the disease causative gene at *GLC1N* [33]. This new approach should enable us to exhaustively search for disease-associated genes in genetic loci.
